# Case Report of Urethral Stenting in a Dog with Multifactorial Lower Urinary Tract Obstruction Associated with Suspected Transitional Cell Carcinoma and Severe Cystolithiasis

**DOI:** 10.3390/vetsci13050472

**Published:** 2026-05-13

**Authors:** Shin-Ho Lee, Jeong-Hyun Seo, Youngkwang Ryu, Jae-Hyeon Cho

**Affiliations:** 1 Department of Companion Animal Health, Tongmyong University, Busan 48520, Republic of Korea; hovet519@tu.ac.kr; 2 Time Animal Medical Center, Daejeon 35233, Republic of Korea; dvmseo@gmail.com (J.-H.S.); yyk4510@naver.com (Y.R.); 3 Institute of Animal Medicine, Gyeongsang National University, Jinju 52828, Republic of Korea

**Keywords:** dog, urethral stent, neoplasia, urolithiasis, hydronephrosis

## Abstract

A 14-year-old spayed female Maltese dog presented with hematuria, pollakiuria, decreased urine volume per voiding, and prolonged urination time, and was diagnosed with multifactorial urinary obstruction involving suspected trigonal neoplasia and extensive urolithiasis affecting the kidneys, ureters, bladder, and urethra. Diagnosis was based on serum biochemical analysis, radiography, and ultrasonography, which revealed diffuse urolithiasis, urethral involvement, and a trigonal mass consistent with a suspected neoplastic lesion. Due to the multifocal nature of obstruction, surgical management was considered impractical. The patient underwent urethral stent placement as a minimally invasive palliative intervention. Following the procedure, rapid restoration of urine flow was achieved, and significant improvement in renal parameters, including blood urea nitrogen and creatinine, was observed within 3 days. During follow-up, the stent remained well positioned without migration. Although transient deterioration, including hydronephrosis and increased renal parameters, was noted at day 52, subsequent improvement was observed by day 64, suggesting a dynamic course of obstruction. This case demonstrates that urethral stenting can be an effective treatment option for managing complex, multifactorial lower urinary tract obstruction in dogs, providing rapid clinical improvement and sustained urinary patency. However, progressive changes in the upper urinary tract may occur, emphasizing the importance of comprehensive evaluation and continuous monitoring.

## Simple Summary

This report describes a dog with complex urinary tract obstruction caused by suspected trigonal neoplasia and extensive urolithiasis affecting multiple levels of the urinary tract. The patient was treated with urethral stent placement to relieve obstruction and restore urine flow. Following the procedure, clinical signs improved rapidly, and renal function showed marked recovery. Although disease progression occurred during follow-up, the stent remained effective in maintaining urinary patency, and overall clinical status improved with continued management. Urethral stenting may be a useful minimally invasive option for managing complicated cases involving both tumor-related and stone-related obstruction, although ongoing monitoring is essential.

## 1. Introduction

Urethral obstruction in dogs is a clinically important condition that can result from a variety of etiologies, including urolithiasis, urethral strictures, and neoplasia. Among neoplastic causes, transitional cell carcinoma (TCC) represents the most common malignant tumor of the canine lower urinary tract and most frequently arises in the trigonal region of the urinary bladder, often leading to urethral involvement and progressive urinary obstruction [1,2]. The management of urethral obstruction depends on the underlying cause, severity, and extent of disease. In cases of malignant obstruction, particularly TCC, urethral stenting has emerged as a minimally invasive palliative treatment option that allows rapid restoration of urine flow and improvement in quality of life [3,4]. Previous studies have demonstrated high technical success rates and effective relief of obstruction following urethral stent placement, making it a widely accepted alternative to more invasive surgical procedures [5,6]. In contrast, urolithiasis is a common cause of lower urinary tract disease in dogs and is typically managed through surgical removal, dissolution therapy, or minimally invasive techniques such as urohydropropulsion or laser lithotripsy [7,8]. However, when calculi are distributed extensively throughout the urinary tract—including the kidneys, ureters, bladder, and urethra—complete resolution may be difficult, and persistent or recurrent obstruction may occur [7,9]. Persistent or recurrent obstruction may also lead to progressive upper urinary tract dilation and renal dysfunction [10].

Although urethral stenting has been well described for both malignant and benign urethral obstruction [3,6], its use in dogs with concurrent, severe, and diffuse urolithiasis affecting multiple levels of the urinary tract has rarely been reported. The coexistence of neoplastic and mechanical obstructive processes presents a unique clinical challenge, as treatment strategies must address both dynamic and structural components of obstruction.

Importantly, even after successful urethral stent placement, progressive upper urinary tract deterioration, including hydronephrosis, may still occur, reflecting the complexity of multifactorial urinary obstruction. The purpose of this report is to describe the clinical findings, therapeutic decision-making, and outcome of urethral stenting in a dog with suspected trigonal neoplasia and severe, multifocal urolithiasis, highlighting the role of urethral stenting in the management of complex, multifactorial lower urinary tract obstruction.

## 2. Case Description

### 2.1. Animal

A 14-year-old spayed female Maltese dog weighing 1.9 kg was referred for evaluation of recurrent hematuria pollakiuria, decreased urine volume per voiding, and prolonged urination time. The patient had a history of urolithiasis diagnosed approximately one year prior and had been managed with a therapeutic diet. Despite dietary management, clinical signs including hematuria and pollakiuria recurred, and progressive enlargement of uroliths was reported by the referring veterinarian. At presentation, the owner reported intermittent hematuria, reduced urine volume per voiding, and prolonged urination time. Physical examination revealed mild dehydration, tachycardia (heart rate 180 bpm), and normal respiratory parameters. Blood analysis showed severe elevations in blood urea nitrogen and creatinine, with both levels significantly exceeding the reference range. Hyperkalemia and hyperphosphatemia were also observed. Initial blood work revealed marked azotemia, characterized by a seven-fold increase in creatinine and a ten-fold increase in BUN, indicating post-renal azotemia secondary to urinary obstruction (Table 1).

### 2.2. Ultrasonography and Blood Test

**Figure 1:**
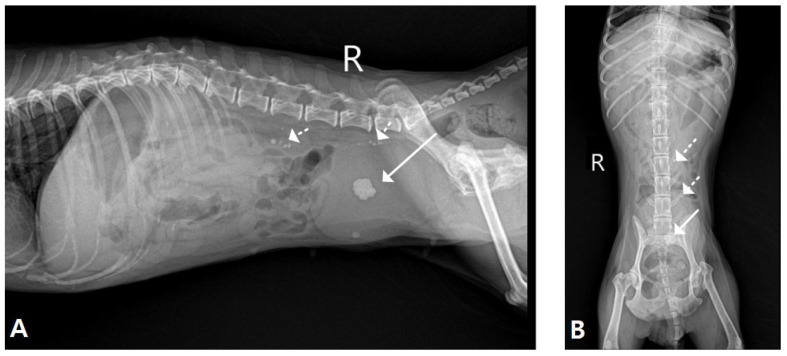
Radiographic findings of the urinary tract. (**A**) Lateral radiographic view showing radiopaque uroliths within the urinary bladder (arrow with line) and distension of the urinary bladder. Additional radiopaque calculi were observed within the kidneys and ureters (arrow with dotted line). (**B**) Ventrodorsal radiographic view demonstrating multiple radiopaque uroliths within the urinary bladder (arrow with line) and calculi distributed along the ureters and kidneys (arrow with dotted line).

**Figure 2:**
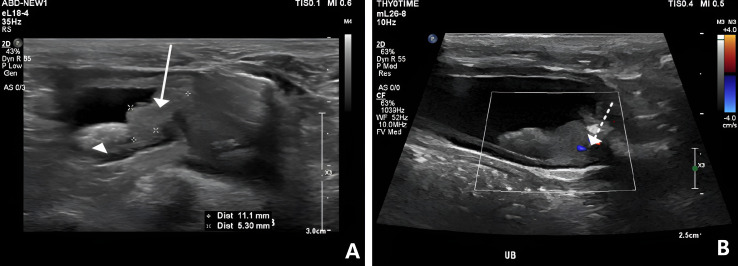
Ultrasonography of a trigonal hyperechoic bladder mass. (**A**) An irregularly marginated hyperechoic nodule (5.3 × 11.1 mm) (arrow with line) and calculi (arrow head) was observed at the urinary bladder trigone. (**B**) The mass contained calcification and exhibits clearly detectable vascular flow (arrow with dotted line).

**Figure 3:**
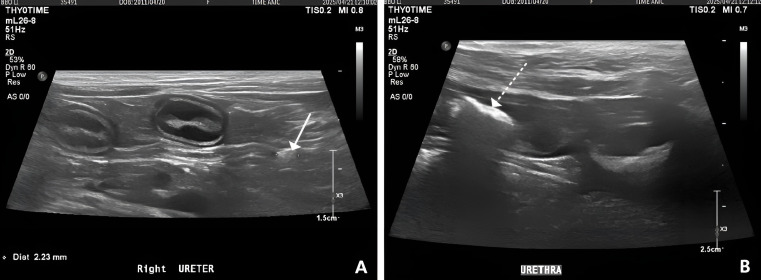
Ultrasonographic images of ureteral and urethral calculi. (**A**) A urolith (arrow with line) measuring approximately 2.23 mm in diameter was identified within the right ureter. (**B**) A urolith (arrow with dotted line) was observed within the left ureter.

Diagnostic imaging was performed, including radiography and ultrasonography. Abdominal radiographs (Figure 1A,B) demonstrated multiple radiopaque calculi distributed throughout the urinary tract, including the kidneys, bilateral ureters, and urinary bladder. Marked distension of the urinary bladder was also observed. Ultrasonographic examination revealed extensive urolithiasis involving both kidneys, ureters, bladder, and urethra. Additionally, a mass measuring approximately 9–12 mm in diameter was identified in the trigonal region of the urinary bladder. The mass exhibited irregular margins, internal vascularization, and partial mineralization, raising strong suspicion for malignant a malignant neoplastic process (Figure 2A,B). Further findings included marked urethral wall thickening and intraluminal urethral, ureteral calculi, suggesting concurrent urethral involvement (Figure 3A,B). Mild dilation of the renal pelvis was observed bilaterally, indicating partial urinary obstruction rather than complete obstruction. Based on imaging findings, the primary differential diagnoses included TCC with urethral invasion, extensive urolithiasis involving the entire urinary tract, and secondary partial urinary obstruction. Although cytologic or histopathologic confirmation was recommended, the owner declined invasive diagnostic procedures at the time of presentation. Given the extensive distribution of calculi and suspected neoplastic involvement of the trigone and urethra, complete surgical management was considered impractical. While cystotomy could have addressed the bladder calculi, concurrent ureteral and urethral calculi, along with probable neoplastic infiltration of the lower urinary tract, significantly limited the likelihood of achieving complete resolution through surgery alone.

Therefore, urethral stenting was selected as a minimally invasive palliative intervention to relieve urethral obstruction and maintain urinary flow. The decision was based on the presence of multifactorial obstruction, including both mechanical obstruction due to calculi and functional obstruction associated with suspected neoplastic disease.

The patient recovered uneventfully from anesthesia. Table 1 presents the serum chemistry and electrolytes results from before the procedure and during day 5. BUN decreased to 46.97 mg/dL and creatinine to 1.38 mg/dL. Potassium levels normalized (3.31 mmol/L), and sodium remained stable within the reference range (146 mmol/L). Phosphorus levels on day 3 were not evaluated. These findings indicated significant improvement in renal function and electrolyte imbalance following stent placement. The observed improvement in renal parameters presented in Table 1 was primarily associated with pre-procedural stabilization, including intravenous fluid therapy and correction of electrolyte imbalances, rather than the immediate effect of urethral stent placement. Prior to the urethral stent placement, the patient underwent initial stabilization, including intravenous fluid therapy and correction of electrolyte imbalances, to improve renal parameters and reduce anesthetic risk. This pre-procedural management contributed to the partial normalization of biochemical abnormalities observed before the intervention.

### 2.3. Urethral Stent Placement Procedure

Urethral stent placement was performed on day 5 of hospitalization after confirmation of stabilization in renal parameters (blood urea nitrogen, creatinine, and phosphorus), in order to minimize anesthetic risk and allow safe induction of general anesthesia. Cefazolin (25 mg/kg, q12h; Cefazolin Inj., Chong Kun Dang Pharm., Seoul, Republic of Korea) was administered as a perioperative prophylactic antimicrobial because the procedure involved urinary tract instrumentation and urethral stent placement under fluoroscopic guidance, which could increase the risk of procedure-related bacterial contamination. Midazolam (0.85 mg/kg, Bukwang Midazolam Inj. Bukwang Pharmcorp., Seoul, Republic of Korea) and propofol (10 mg/kg, Provive, Myungmoon Pharm, Seoul, Republic of Korea) were administered for an induction procedure. After intubation using an endotracheal tube (Rushelit, size ID 3.5 mm, OD 5.3 mm, Teleflex, Seoul, Republic of Korea ), general anesthesia using isoflurane (Ifran, Hana Pharm, Seoul, Republic of Korea) was induced by forced breathing circulation using a respiratory anesthetic machine (Drager primus, Dragerwerk AG & Co. KGaA, Lübeck, Germany) with a volume of 50 to 60 cc under general anesthesia with using a C-arm system (OSCAR 15, Genoray Co., Ltd., Seoul, Republic of Korea) to visualize contrast imaging and monitor the urethral stent (Urethra Stent 4–30 mm, PNG MEDTEK Inc., Seoul, Republic of Korea) placement procedure in real time. The patient was positioned in ventral recumbency, and the perineal region was aseptically prepared. Continuous monitoring of vital parameters, including heart rate, respiratory rate, and oxygen saturation, was maintained throughout the procedure.

A retrograde urethrogram was initially performed to delineate the urethral lumen and to identify the location, length, and severity of the obstruction. Contrast medium (OMNIPAQUE^TM^, GE HealthCare, Chicago, IL, USA) was infused via a urinary catheter, which demonstrated marked urethral narrowing at the level of the trigonal region and proximal urethra, consistent with intraluminal obstruction.

Following contrast evaluation, a hydrophilic guidewire was advanced through the urethra under fluoroscopic guidance. In female dogs, the urethra was relatively short and wide, with a mild curvature at the proximal urethra near the trigonal region. Resistance was encountered during guidewire advancement at this level, and passage through the stenotic segment required careful manipulation. The guidewire was successfully advanced across the obstruction and positioned within the urinary bladder.

Over the guidewire, a vascular sheath or delivery system was introduced, and urethral measurements were obtained fluoroscopically to determine the appropriate stent diameter and length. A self-expanding metallic stent was selected to cover the entire length of the stenotic segment, including adequate proximal and distal margins.

The stent delivery system was advanced over the guidewire to the target site under continuous fluoroscopic visualization. Positioning was confirmed using contrast injection and anatomical landmarks. The stent was then gradually deployed, allowing controlled expansion across the stenotic urethral segment.

Post-deployment fluoroscopic imaging confirmed appropriate stent expansion, accurate positioning, and restoration of urethral patency. A repeat contrast study demonstrated improved urine flow without evidence of leakage or immediate complications. The guidewire and delivery system were subsequently removed, and spontaneous urine flow through the urethra was observed. Supplementary Video S1. Fluoroscopy-guided urethral stent placement in the present case. The video shows retrograde contrast evaluation, guidewire passage through the stenotic segment, and deployment of a self-expanding metallic stent under real-time C-arm fluoroscopic guidance.

Mild hematuria was observed at the end of urination following the procedure; however, urine output was within normal limits. Although assisted feeding was required, vital signs remained stable, and no vomiting or diarrhea was observed. The patient was discharged with a 7-day course of medications. Management of anti-infection and pain control was conducted with amoxicillin, clavulanic acid (20 mg/kg, q12 h, Amocla, KUHNIL Corp., Seoul, Republic of Korea), omeprazole (1 mg/kg, q12h, Omed Tab, SK Chemicals, Seoul, Republic of Korea), and gabapentin (5 mg/kg, q12 h, Gabalep Cap., Chong Kun Dang Pharmaceutical Corp., Seoul, Republic of Korea).

### 2.4. Post-Procedural Follow-Up

**Figure 4:**
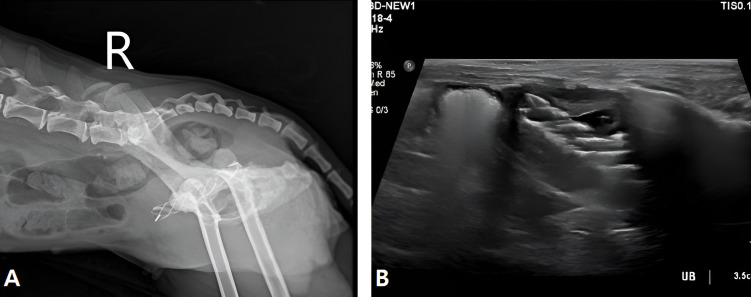
Post-procedural findings following urethral stent placement. (**A**) Lateral radiographic image showing appropriate positioning of the urethral stent. (**B**) Ultrasonographic image demonstrating the stent within the urethral lumen with maintained patency.

On day 6 following the procedure, the urethral stent remained well positioned, with no evidence of migration or displacement on radiographic evaluation (Figure 4A). Approximately 1.1 cm of luminal patency was secured beyond the bladder trigonal mass. Ultrasonographic examination confirmed that the cranial margin of the stent was in contact with the bladder wall (Figure 4B). No signs of urinary incontinence were observed. Although the duration of urination remained slightly prolonged relative to perceived normal, both voided urine volume per episode and overall voiding efficiency had improved compared to pre-procedural status. No additional abnormalities were identified during follow-up.

On day 52 following the procedure, the patient presented with persistent mild hematuria that had been observed for approximately three days, although intermittent normal urination had been noted previously. Clinical signs included pollakiuria, and urinary incontinence. A small amount of mucus-like material was noted in the urine one day prior to evaluation. Appetite had decreased, with near-complete anorexia since the previous day. The patient was fed a renal diet consisting of Royal Canin Renal Dogs^®^ (Royal Canin, Aimargues, France) and Hill’s Prescription Diet^®^ (Hill’s Pet Nutrition, Overland Park, KS, USA), and partial intake was observed only when mixed with rice. No vomiting or diarrhea was reported; however, decreased activity and reduced water intake were noted compared to the previous condition.

Blood analysis revealed elevated renal parameters, including blood urea nitrogen (85.01 mg/dL), creatinine (2.68 mg/dL), and phosphorus (8.4 mg/dL), indicating deterioration of renal function.

**Figure 5:**
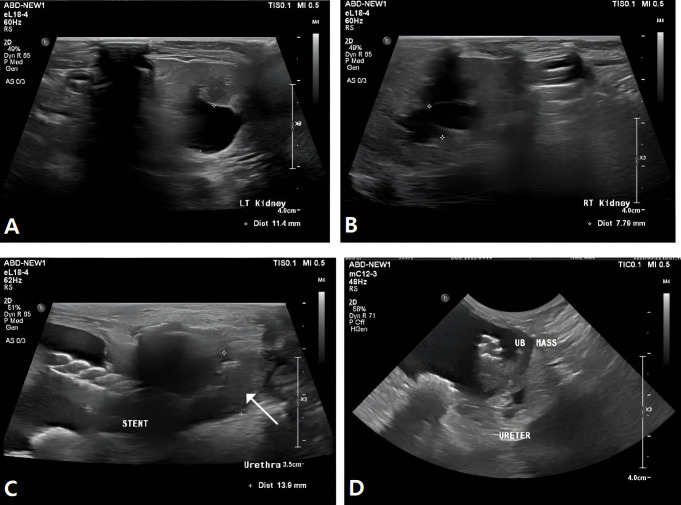
Follow-up ultrasonographic findings demonstrating disease progression and bilateral hydronephrosis. (**A**) Ultrasonographic image of the left kidney showing renal pelvic dilation (11.4 mm), consistent with hydronephrosis. (**B**) Ultrasonographic image of the right kidney demonstrating renal pelvic dilation (7.7 mm). (**C**) Ultrasonographic image showing tumor invasion (arrow with line) into the urethra distal to the caudal margin of the urethral stent. (**D**) Ultrasonographic image demonstrating marked progression of the mass, with increased thickness up to 24 mm.

Ultrasonographic examination of the urinary tract revealed bilateral hydronephrosis, with renal pelvic dilation measuring 11.4 mm in the left kidney and 7.7 mm in the right kidney (Figure 5A,B). Due to the presence of hydronephrosis and suspected urinary outflow impairment, ultrasound-guided cystocentesis was performed.

The urethral stent remained well positioned, maintaining approximately 1.1 cm of luminal patency beyond the bladder trigonal mass, with no evidence of migration. However, tumor invasion into the urethra was identified distal to the stent, extending approximately 2.0 cm caudally from the stent margin (Figure 5C). In addition, marked tumor progression was observed, with an increase in mass thickness up to approximately 24 mm (Figure 5D).

**Figure 6:**
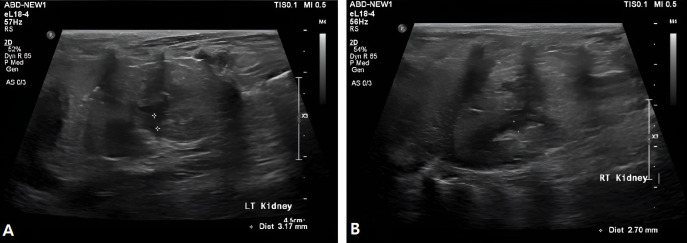
Ultrasonography of bilateral renal pelvis. (**A**) Ultrasonographic image of the left kidney demonstrating renal pelvic dilation measuring 3.17 mm. (**B**) Ultrasonographic image of the right kidney demonstrating renal pelvic dilation measuring 2.70 mm.

On day 64 following the procedure, blood analysis showed improvement in renal parameters, with blood urea nitrogen decreased to 79.57 mg/dL, creatinine to 1.65 mg/dL, and phosphorus to 4.53 mg/dL. Ultrasonographic evaluation demonstrated marked reduction in renal pelvic dilation, with measurements of 3.2 mm in the left kidney and 2.7 mm in the right kidney (Figure 6A,B). Mild hematuria was still observed at the beginning of urination; however, urine color returned to normal thereafter, and overall urine volume was considered adequate. Appetite was also reported to be good.

## 3. Discussion

This case describes urethral stent placement in a dog with suspected trigonal neoplasia and extensive, multifocal urolithiasis involving multiple levels of the urinary tract. The coexistence of these conditions resulted in a complex, multifactorial urinary obstruction, involving both mechanical obstruction from calculi and functional obstruction associated with suspected neoplastic disease. TCC is the most common malignant tumor of the canine lower urinary tract and most frequently involves the trigonal region, often leading to urethral invasion and progressive urinary obstruction [2]. Due to its anatomical location and infiltrative behavior, complete surgical excision is rarely feasible, and treatment is typically focused on palliation and maintenance of urinary outflow [11].

In addition to suspected neoplasia, this patient exhibited severe and diffuse urolithiasis involving the kidneys, ureters, bladder, and urethra. Although urolithiasis is a common condition in dogs, widespread distribution across multiple anatomical levels presents a significant therapeutic challenge. In such cases, complete removal of calculi is often impractical, and persistent or recurrent obstruction may occur due to both physical blockage and secondary functional alterations within the urinary tract [7].

The present case highlights the clinical significance of multifactorial obstruction. When both neoplastic and mechanical components coexist, conventional surgical approaches are often insufficient. In this patient, the presence of ureteral and urethral calculi, combined with suspected trigonal involvement, limited the feasibility of surgical intervention and supported the decision to pursue a minimally invasive palliative approach. Urethral stenting has been well established as an effective minimally invasive technique for relieving urethral obstruction in dogs, particularly in cases of malignant disease [3]. Although the imaging findings in this case strongly suggested TCC, alternative diagnoses should be considered, particularly in the absence of cytological or histopathological confirmation. Differential diagnoses include non-neoplastic conditions such as polypoid cystitis, granulomatous inflammation, and reactive urothelial hyperplasia, all of which may present as mass-like lesions in the urinary bladder and mimic neoplastic processes on ultrasonography [2,11]. Therefore, the diagnosis of TCC in this case should be interpreted as presumptive rather than definitive. Previous studies have shown that canine TCC is generally not curable due to its infiltrative nature and typical location within the trigonal region of the urinary bladder; however, it is often clinically manageable for several months with maintenance of acceptable quality of life [11]. Reported outcomes indicate that many dogs respond favorably to medical management, and overall survival is commonly less than one year, although prognosis varies depending on tumor location, extent of urinary obstruction, presence of metastasis, and treatment modality [3,4,11]. Accordingly, the goal of urethral stenting in the present case was not curative treatment, but palliation of urinary obstruction and stabilization of the patient’s clinical condition. In the present case, urethral stent placement resulted in rapid restoration of urine flow and was associated with marked improvement in renal parameters, including a significant decrease in blood urea nitrogen and creatinine concentrations within 3 days.

Post-renal azotemia results from urinary outflow obstruction, leading to reduced glomerular filtration rate and electrolyte imbalance. Relief of obstruction can result in rapid improvement in renal function if intervention is performed before irreversible renal damage occurs [12]. The normalization of potassium levels observed in this case further supports the resolution of obstructive uropathy following stent placement. Despite the initial clinical improvement, bilateral hydronephrosis developed at day 52, accompanied by worsening renal parameters.

Despite the initial clinical improvement, bilateral hydronephrosis developed at day 52, accompanied by worsening renal parameters. The dynamic clinical course observed in this case, characterized by initial improvement, subsequent deterioration, and partial recovery, may be explained by the complex interaction between mechanical and functional components of urinary obstruction. Urolithiasis likely contributed to intermittent ureteral obstruction, while suspected neoplastic involvement may have caused progressive and variable compression of the urinary tract. These combined factors could lead to fluctuating renal function and transient hydronephrosis depending on the degree and location of obstruction [10,12].

This finding suggests that urethral stenting alone may not fully prevent progressive upper urinary tract deterioration in cases of multifactorial obstruction. Potential contributing factors include continued tumor progression, secondary ureteral obstruction due to calculi, and functional impairment of urinary drainage. Importantly, urethral stenting primarily addresses distal urinary outflow obstruction and may be insufficient in cases with concurrent upper urinary tract involvement. Therefore, in patients with diffuse urolithiasis or suspected ureteral obstruction, stent placement should be considered as part of a broader management strategy rather than a definitive solution.

Interestingly, partial resolution of hydronephrosis and improvement in renal parameters were observed at day 64, indicating that the degree of obstruction in such cases may be dynamic rather than static. This fluctuation may reflect transient changes in ureteral patency, movement of calculi, or variations in tumor-related compression. Although urethral stenting is effective, it is associated with potential complications, including urinary incontinence, stent migration, and recurrent obstruction [3]. In cases involving the trigonal region, the risk of urinary incontinence may be increased due to disruption of urethral sphincter function. However, in this case, no significant urinary incontinence was observed, suggesting that careful placement and monitoring can minimize such complications.

A limitation of this report is the absence of histopathologic confirmation of the suspected neoplasm. Although imaging findings strongly suggested TCC, a definitive diagnosis could not be established. Additionally, the follow-up period was relatively limited, restricting evaluation of long-term oncologic outcome, survival time, and stent durability.

To the authors’ knowledge, this case represents a rare presentation of urethral stent placement in a dog with concurrent suspected trigonal neoplasia and extensive, multifocal urolithiasis affecting multiple levels of the urinary tract. This case highlights the clinical utility of urethral stenting as an effective palliative option, while also emphasizing the importance of comprehensive evaluation and ongoing monitoring in patients with complex, multifactorial urinary obstruction.

## 4. Conclusions

Urethral stenting provided rapid relief of urinary obstruction and resulted in significant improvement in renal function in a dog with suspected trigonal neoplasia and extensive urolithiasis. This technique represents a valuable minimally invasive palliative option for managing complex, multifactorial lower urinary tract obstruction. In addition, this case highlights that favorable clinical outcomes can be achieved even in challenging cases involving both neoplastic and mechanical obstruction. Careful monitoring and comprehensive evaluation of the urinary tract may further support sustained clinical improvement.

## Figures and Tables

**Table 1:** Changes in renal function markers over 5 Days.

Test Item	Day	Measured Value	Reference Range
Blood Urea Nitrogen	Day 1 of hospitalization	180.76 mg/dL	8–26 mg/dL
	Day 3 of hospitalization	46.97 mg/dL	
	Day 5 of hospitalization	30.03 mg/dL	
Creatinine	Day 1 of hospitalization	7.24 mg/dL	0.5–1.3 mg/dL
	Day 3 of hospitalization	1.38 mg/dL	
	Day 5 of hospitalization	1.21 mg/dL	
Phosphorus	Day 1 of hospitalization	17.4 mg/dL	3–6.2 mg/dL
	Day 3, 5 of hospitalization	-	
Na	Day 1 of hospitalization	146 mmol/L	145–151 mmol/L
	Day 3 of hospitalization	146 mmol/L	
	Day 5 of hospitalization	146 mmol/L	
K	Day 1 of hospitalization	6.9 mmol/L	3.9–5.1 mmol/L
	Day 3 of hospitalization	3.31 mmol/L	
	Day 5 of hospitalization	4.08 mmol/L	

## Data Availability

The original contributions presented in this study are included in the article/Supplementary Materials. Further inquiries can be directed to the corresponding author(s).

## Supplementary Materials

The following supporting information can be downloaded at: https://www.mdpi.com/article/10.3390/vetsci13050472/s1, Video S1: Urethral stent placement under fluoroscopic guidance. Real-time fluoroscopic visualization of guidewire passage and stent deployment across the stenotic urethra.
